# Pregnancy After Breast Cancer: A Narrative Review of Oncologic Safety, Obstetric and Neonatal Outcomes, and Reproductive Survivorship

**DOI:** 10.3390/cancers18162715

**Published:** 2026-08-21

**Authors:** Vahideh MoghaddamHosseini, Afshin Zand

**Affiliations:** 1 Non-Communicable Diseases Research Center, Sabzevar University of Medical Sciences, Sabzevar 9613873137, Iran; moghaddamvahideh@gmail.com; 2 Department of Public Health Medicine, Medical School, University of Pécs, 7624 Pécs, Hungary

**Keywords:** breast cancer, pregnancy, oncofertility, fertility preservation, obstetric outcomes, neonatal outcomes, survivorship

## Abstract

**Background:** Survival after early breast cancer has improved to the point where reproductive health is no longer a peripheral concern in young survivors but a routine one. This review synthesizes current evidence on oncologic safety, obstetric and neonatal outcomes, fertility preservation, breastfeeding, and psychosocial survivorship to develop a counseling framework that clinicians can apply in practice. **Methods:** This narrative review was informed by a structured literature search of PubMed/MEDLINE, Embase, and the Cochrane Library covering studies published from January 2000 to July 2026, with greater analytic weight given to studies published from 2010 onward to ensure the synthesis reflects contemporary treatment standards. Relevant evidence was identified and narratively synthesized according to the clinical and reproductive aspects of pregnancy after breast cancer. No formal risk-of-bias assessment or quantitative synthesis was undertaken. **Results:** On the currently available evidence, pregnancy after breast cancer is not associated with an increased risk of recurrence. This holds in hormone receptor-positive disease and in *BRCA1/2* carriers, and it is supported prospectively by the POSITIVE trial, whose preplanned update at a median follow-up of 71 months found a five-year incidence of breast cancer-free interval events of 12.3% against 13.2% in matched external controls. Obstetric and neonatal risks are higher than in the general obstetric population, although the pooled effect sizes are small to moderate: odds ratios are approximately 1.14 for cesarean delivery, 1.45 for preterm birth, 1.50 for low birth weight, and 1.16 for small-for-gestational-age birth. The excess concentrates in pregnancies conceived within the first one to two years after diagnosis and attenuates thereafter. Fertility preservation techniques appear both effective and oncologically safe. Fertility-related distress, by contrast, is common and does not reliably resolve once treatment ends. **Conclusions:** Oncologic risk alone is not a reason to advise against pregnancy after breast cancer. Early oncofertility referral, multidisciplinary planning, and risk-adapted obstetric surveillance remain the priorities. Because the evidence base is predominantly observational, these conclusions describe what the current data show rather than establishing safety definitively.

## Simple Summary

Treatment has improved to the point where most young women diagnosed with breast cancer will survive it, and many of them still hope to have children. Whether pregnancy is safe afterwards remains one of the questions they ask most often, and one that clinicians have historically answered with caution rather than with data. This review draws together the current literature across five areas: the risk that the cancer returns, complications during pregnancy and delivery, the health of the infant, the options for preserving fertility before treatment begins, and the emotional weight that reproductive decisions carry for survivors. On the evidence available so far, the older assumption that pregnancy is hazardous is not supported. Women who conceive after breast cancer do not appear to relapse more often, and their infants are not born with more birth defects. What does appear to matter is timing: the risk of preterm birth and low birth weight is highest when conception occurs soon after diagnosis, particularly within the first year, and falls away as the interval lengthens. This is a factor to weigh alongside tumor biology and treatment history rather than a fixed waiting period that applies to everyone. The practical message for clinicians is that fertility belongs in the conversation at diagnosis, not after treatment has ended.

## 1. Introduction

Breast cancer is increasingly diagnosed in women who have not yet passed childbearing age, which has moved fertility and pregnancy from the periphery of survivorship care toward its center [1,2,3,4,5]. Registry data from global and United States sources describe the same trend: incidence in women of reproductive age is rising across most world regions and across racial and ethnic groups, with the sharpest increases in the younger age strata and in estrogen receptor-positive disease [2,5,6]. Reproductive planning after treatment has therefore become a routine rather than an exceptional part of follow-up. Two anxieties dominate these consultations. The first is that the hormonal environment of pregnancy might reactivate a hormone-sensitive tumor, a concern with a long history in the literature [1,7,8,9,10]; as discussed below, it has not held up well against accumulating evidence. The second is a genuine constraint rather than a perception: chemotherapy is gonadotoxic, and five to ten years of adjuvant endocrine therapy consume precisely the years in which conception would otherwise be attempted [1,4,11,12]. Its consequences are measurable, in that breast cancer survivors are considerably less likely than women of the same age in the general population to achieve a subsequent pregnancy [9,10]. Fertility concern also shapes treatment itself, with some women reporting that worry about future childbearing influenced the therapy they were willing to accept [13,14].

Professional guidance has, in several respects, moved faster than routine practice. Fertility counseling is now treated as a standard component of cancer care rather than an optional adjunct, with recommendations to raise reproductive risk at the time of diagnosis and to refer to a reproductive specialist before treatment begins wherever the schedule allows [10,15,16]. Embryo cryopreservation, oocyte cryopreservation, and ovarian tissue cryopreservation are all recognized as established options, and current guidance places as much emphasis on the multidisciplinary pathway as on the technique itself [13,17].

Recent reviews have tended to address oncologic safety in isolation, or fertility preservation in isolation. The present review takes a wider frame, bringing together oncologic safety, obstetric and neonatal outcomes, fertility preservation, breastfeeding, and the psychosocial dimensions of reproductive decision-making, on the argument that women weigh these domains simultaneously and that clinicians are asked about all of them within the same consultation.

## 2. Materials and Methods

This is a narrative review supported by a structured literature search to improve transparency and reproducibility; it was not conducted as a systematic review. PubMed/MEDLINE, Embase, and the Cochrane Library were searched from 1 January 2000 to 1 July 2026. The full strategy combined controlled vocabulary with free-text terms in the form: (“breast neoplasms”[MeSH] OR “breast cancer”) AND (pregnan* OR “reproductive health” OR “obstetric outcome*” OR “neonatal outcome*” OR oncofertility OR “fertility preservation” OR survivorship), adapted to the syntax of each database. Reference lists of included articles and of relevant guidelines were hand-searched for additional records. Peer-reviewed clinical studies, systematic reviews, meta-analyses, and clinical practice guidelines were prioritized. Synthesis was qualitative and deliberately cross-disciplinary, drawing on oncology, reproductive endocrinology, maternal–fetal medicine, and psychosocial oncology. Complete database-specific search strategies are provided in Supplementary Table S1. The 2000 start date was chosen because it marks the point at which taxane-based chemotherapy, trastuzumab, and modern endocrine schedules entered routine practice, and because the large international cohorts that underpin this field recruit from that year onward. We recognize that a window of this length inevitably captures treatment eras that no longer reflect current standards. Two steps were taken to limit the resulting distortion. First, analytic weight was placed on studies published from 2010 onward, and pre-2010 work is cited only where it retains historical or methodological relevance, such as the early matched analyses that first addressed the healthy mother effect. Second, where older and newer evidence conflict, the more recent estimate is reported in the text and the discrepancy is discussed rather than averaged away.

Records were exported to EndNote 2025.3 software, where duplicates were removed electronically and the remainder checked by hand. Screening and data extraction were performed independently by two reviewers using standardized extraction forms, with disagreements resolved by discussion. No review protocol was prospectively registered, and no formal risk-of-bias instrument, such as ROBINS-I or the Newcastle–Ottawa Scale, was applied. This reflects the review’s narrative design. In place of formal appraisal, each included study was characterized by its design, sample size, length of follow-up, comparator, and the extent to which the healthy mother effect had been addressed through matching or adjustment; these characteristics are reported in Table 1 and used throughout the text to weigh competing findings. Prospective and adequately matched studies are given precedence over unmatched retrospective series wherever the two disagree.

Eligible studies reported pregnancy outcomes in premenopausal women whose breast cancer had been diagnosed before conception. Pregnancy-associated breast cancer, diagnosed during gestation or within one year postpartum, was excluded, as were case reports, case series of fewer than ten patients, editorials, and publications not available in English. Studies of women who received chemotherapy during an established pregnancy were not treated as evidence about conception after treatment; where such work is cited, in Section 3.4, it is labeled explicitly as indirect evidence bearing on a different clinical question.

During the preparation of this manuscript, the authors used a generative artificial intelligence tool for language editing and stylistic revision of author-drafted text. The authors have reviewed and edited the output and take full responsibility for the content of this publication.

## 3. Results

### 3.1. Oncologic Safety: Does Pregnancy Increase the Risk of Recurrence?

Few questions in survivorship care have been examined as repeatedly, and the accumulated answer is reassuring [11,17,23,24]. A 2021 systematic review and meta-analysis of 39 studies, covering 112,840 women with breast cancer of whom 7505 conceived after diagnosis, reported that women who conceived after breast cancer had better disease-free (HR 0.66, 95% CI 0.49–0.89) and overall survival (HR 0.56, 95% CI 0.45–0.68) than those who did not; the finding survived adjustment for confounders and held across tumor and treatment subgroups [24]. A 2025 meta-analysis pooling 52 studies (9288 women with a pregnancy after diagnosis and 123,429 without) reached the same conclusion, reporting improved overall survival (RR 1.09, 95% CI 1.06–1.12; *p* < 0.001) alongside reductions in any recurrence (RR 0.61, 95% CI 0.55–0.68; *p* < 0.001) and in distant recurrence (RR 0.50, 95% CI 0.37–0.68; *p* < 0.001), independent of stage, receptor status, and treatment received. *BRCA2* mutation appeared to attenuate the disease-free survival advantage, but pregnancy in hormone receptor-positive disease carried no prognostic penalty [11]. It is worth being precise about what these pooled figures can and cannot establish. All of the contributing studies are non-randomized, so the direction of the association is secure, but its magnitude is not, and a hazard ratio below unity for survival should be read as evidence against harm rather than as evidence of benefit.

Retrospective cohorts have largely converged on the same position [14,23,25]. A multicenter matched analysis found no difference in disease-free survival between women who became pregnant and those who did not, in either ER-positive or ER-negative disease, although overall survival again favored the pregnancy group [7]. Earlier matched and adjusted series had likewise reported no significant excess of recurrence or death following post-treatment pregnancy [14,25].

The evidence in hormone receptor-positive disease, historically the thinnest, has improved substantially. A systematic review and meta-analysis of 3805 women with prior hormone receptor-positive early breast cancer (1285 of whom conceived, with median follow-up across the contributing studies ranging from 3.8 to 15.8 years) found no difference in disease-free survival between those who conceived and those who did not (HR 0.96, 95% CI 0.75–1.24; *p* = 0.781), with better overall survival in the pregnancy group (HR 0.46, 95% CI 0.27–0.77) [1]. The question carries disproportionate weight in this population because adjuvant endocrine therapy runs for five to ten years and pregnancy during active treatment is contraindicated [1,12]. The POSITIVE trial addressed the resulting impasse directly and prospectively. Among 516 women aged 42 years or younger with stage I–III hormone receptor-positive disease who had completed 18–30 months of endocrine therapy, temporary interruption to attempt conception yielded a pregnancy rate of 74.0% and a live birth rate of 63.8%. Over 1638 patient-years of follow-up, breast cancer events remained below the prespecified safety threshold, with a three-year incidence of 8.9% against 9.2% in the external control cohort [26].

Since that first report, a preplanned analysis with a further 2.5 years of observation has been published, and it strengthens the case considerably [27]. At a median follow-up of 71 months in POSITIVE and 80 months in the SOFT/TEXT cohorts used as bootstrap-matched external controls, the five-year cumulative incidence of breast cancer-free interval events was 12.3% in POSITIVE and 13.2% in SOFT/TEXT (difference −0.9%, 95% CI −4.2% to 2.6%). Distant recurrence-free interval events occurred in 6.2% and 8.3%, respectively (difference −2.1%, 95% CI −4.5% to 0.4%). Of the 497 women followed for non-disease outcomes, 377 (76%) had at least one documented pregnancy on trial, and 343 (69%) had at least one live birth, resulting in 440 offspring. Among the 180 women (36%) who had undergone embryo or oocyte cryopreservation before enrolment, the five-year incidence of breast cancer-free interval events was 14.0% (95% CI 9.6–20.2) compared with 11.5% (95% CI 8.4–15.7) in those who had not, a difference well within the play of chance in a subgroup of this size but one that argues for continued surveillance rather than for reassurance [27].

These are the longest prospective data available, and they should still be read within their limits. POSITIVE is single-arm, so the comparison rests on external controls drawn from trials with different eligibility criteria and a different treatment era. The enrolled population was selected: participants were young, motivated to conceive, had tolerated 18–30 months of endocrine therapy without recurrence, and were overwhelmingly stage I or II, with only a small minority carrying the nodal burden that defines high-risk disease. Women who relapsed during the first two years of endocrine therapy never entered the trial, which is a form of survivorship selection that no statistical adjustment can undo.

Nor does a median follow-up approaching six years settle the question for hormone receptor-positive disease, where the recurrence hazard persists for fifteen years and beyond; follow-up is planned to continue to 2029. POSITIVE therefore supports temporary interruption of endocrine therapy as a reasonable option for carefully selected women, discussed within a multidisciplinary team. It does not license the practice in women with high-risk residual disease, and it should not be presented to patients as a demonstration that interruption is risk-free.

*BRCA1/2* carriers, who face a distinct combination of reproductive and oncologic considerations, show a comparable pattern [18,28]. In the 2020 international cohort of 1252 carriers diagnosed at 40 years or younger, 195 women conceived after diagnosis, giving a cumulative incidence of pregnancy at ten years of 19% (95% CI 17–22%). Disease-free survival did not differ between those who conceived and those who did not (adjusted HR 0.87, 95% CI 0.61–1.23; *p* = 0.41) [28]. The larger 2024 international hospital-based cohort recruited 4732 carriers from 78 centers worldwide, of whom 659 had at least one pregnancy after breast cancer and 4073 did not. The cumulative incidence of pregnancy at ten years was 22% (95% CI 21–24%), with a median time from diagnosis to conception of 3.5 years (IQR 2.2–5.3). Over a median follow-up of 7.8 years (IQR 4.5–12.6), disease-free survival was again equivalent (adjusted HR 0.99, 95% CI 0.81–1.20) [18].

Two features of the 2024 cohort deserve more attention than they usually receive. The first is the divergence by gene. In exploratory analyses, disease-free survival after pregnancy was more favorable in *BRCA1* carriers (adjusted HR 0.80) and less favorable in *BRCA2* carriers (adjusted HR 1.55), a split that echoes the attenuated advantage reported for *BRCA2* in the 2025 meta-analysis [11,18]. These are subgroup estimates from a retrospective cohort, unadjusted for multiplicity, and they are not a basis for advising *BRCA2* carriers against pregnancy. They do justify saying, in counseling, that the reassurance is better established for *BRCA1* than for *BRCA2*, and that this is an area of genuine uncertainty. The second is that obstetric and fetal outcomes in the carrier cohort were unremarkable: of 517 completed pregnancies, 406 (91.0%) delivered at term, 54 (10.4%) were twin pregnancies, induced abortion and miscarriage occurred in 6.9% and 9.7% respectively, and congenital anomalies were documented in four of 470 infants in the available data (0.9%), figures consistent with those expected in women of similar age without a cancer history [18].

Carriers also face decisions that non-carriers do not, and these interact with timing. Risk-reducing salpingo-oophorectomy is generally recommended between 35 and 40 years of age for *BRCA1* carriers and between 40 and 45 for *BRCA2* carriers, which compresses the reproductive window and makes early fertility preservation more, not less, important. Preimplantation genetic testing for monogenic disease is an option some carriers wish to discuss, and it requires embryo rather than oocyte cryopreservation, a distinction that has to be raised before treatment starts rather than afterwards. Adjuvant olaparib, now standard for high-risk carriers after chemotherapy, adds a further year to the interval before conception can be attempted.

These findings should be read with their principal weakness in view. Most of the underlying studies are observational, and observational designs in this setting are vulnerable to the healthy mother effect: women who go on to conceive tend to be those whose disease and general health already looked favorable, which inflates the apparent survival benefit [13,14,19]. Several investigators mitigated the problem by matching on disease-free interval or on recurrence status at conception, though residual confounding cannot be dismissed [7,14,28]. The apparent survival advantage should therefore not be reported to patients as a benefit of pregnancy. The most defensible reading is that the pooled estimates exclude a clinically meaningful increase in recurrence risk, and that the point estimates favoring the pregnancy group are at least partly an artifact of who becomes pregnant. POSITIVE carries more weight precisely because it was prospective [26,27], and its updated analysis extends the safety window to five years [29], but even 71 months of median follow-up is short relative to the late recurrence pattern characteristic of hormone receptor-positive disease. The proposition supported by the current literature is therefore narrower than the word “safe” implies: pregnancy after breast cancer has not been shown to increase recurrence risk in the populations studied so far, over the follow-up available to date. That is a conclusion about the state of the evidence, not a demonstration of safety, and it remains provisional in higher-risk subgroups and in women treated with newer adjuvant agents.

### 3.2. Obstetric Outcomes in Breast Cancer Survivors

Pregnancy after breast cancer is achievable, but it is not statistically common [4,30,31]. Conception itself is less likely: pooled estimates indicate roughly a 40% lower probability of subsequent pregnancy compared with age-matched women without a breast cancer history (RR 0.40, 95% CI 0.32–0.49) [24,32]. Among women who do conceive, the 2021 meta-analysis identified higher risks of cesarean delivery (OR 1.14, 95% CI 1.04–1.25), preterm birth (OR 1.45, 95% CI 1.11–1.88), low birth weight (OR 1.50, 95% CI 1.31–1.73), and small-for-gestational-age (SGA) birth (OR 1.16, 95% CI 1.01–1.33), with prior chemotherapy exposure the most consistent predictor [24]. These are the estimates that should anchor counseling, and their scale is worth stating plainly: they correspond to relative increases of roughly 14% to 50%, not to the several-fold elevations that the interval-specific analyses discussed below can suggest when read in isolation.

The interval between diagnosis and conception emerges repeatedly as the modifier that matters most. In a Swedish national cohort of 926 births matched against 92,490 comparator births, conception within the first year after diagnosis was associated with elevated risks of cesarean section (RR 1.7), very preterm birth (RR 5.3), and low birth weight (RR 2.7); beyond two years, outcomes approached those of the comparison group [20]. A German database study of 74 survivors and 222 controls found an increased risk of SGA birth overall (OR 3.24, 95% CI 1.17–8.97), rising further with delivery within two years of diagnosis for both SGA (OR 5.73) and low birth weight (OR 4.57) [21]. The German estimates rest on a small number of exposed pregnancies, and the confidence intervals are correspondingly wide [18]. The pattern across both cohorts is a gradient rather than a step. Risk is highest in the first year, falls appreciably in the second, and by the third is difficult to distinguish from that of the comparison population. Reading this as a two-year rule imposes a threshold that the data do not contain, and we have avoided that framing throughout. The interval since diagnosis is one risk-modifying variable among several, to be weighed against stage, tumor biology, nodal status, the specific agents received, the duration of planned endocrine or targeted therapy, age and ovarian reserve, and how much reproductive time a given woman actually has. For a 39-year-old with node-negative, hormone receptor-negative disease and declining ovarian reserve, the balance may favor conceiving at eighteen months; for a 29-year-old with high-risk node-positive disease on abemaciclib, it may favor waiting well beyond two years.

Counseling drawn from these figures should separate relative from absolute risk. A relative risk of 5.3 for very preterm birth is arresting; very preterm birth is also uncommon, occurring in roughly 1% of deliveries in most European populations, so a fivefold increase moves the absolute probability into the region of 5%, and leaves a 95% chance that it does not occur. The absolute probability that a given survivor delivers a healthy infant at term nonetheless remains high, and the great majority of pregnancies in these cohorts ended in healthy live births [18]. Large delivery datasets make the same point from a different direction: survivors show higher frequencies of fetal distress, preterm labor, cesarean section, and maternal infection, yet absolute event rates stay low [17]. Neither metric is sufficient on its own. Relative risks are what the literature reports and what makes clinical differences visible, and they are the appropriate currency for comparing subgroups or judging whether an association exists at all; absolute risks are what patients actually experience and what determines whether a difference is worth acting on. We have reported relative estimates with their confidence intervals throughout, and paired them with the corresponding absolute frequencies wherever the source data allow.

The most recent synthesis extends the list of signals rather than altering its shape. The 2025 reproductive-outcome meta-analysis confirmed lower pregnancy prevalence, lower childbirth rates, and higher rates of cesarean delivery and preterm birth, and additionally detected associations with intrapartum hemorrhage, induced delivery, prolonged labor, fetal stress, and delivery-related complications [4].

Not every cohort finds the same magnitude of effect. In a French series of 133 survivors contributing 197 pregnancies, previous exposure to chemotherapy, endocrine therapy, or trastuzumab showed no significant association with obstetric or neonatal outcomes, which were broadly comparable to those of the general obstetric population [22]. Several features of that study explain the discrepancy without requiring the underlying biology to differ. With 197 pregnancies and event rates in the range of 5–10%, the series had limited power to detect odds ratios of the size the meta-analyses report; an OR of 1.45 for preterm birth would need several hundred exposed pregnancies to reach conventional significance. The cohort was also drawn from a single tertiary center with a longer median interval from diagnosis to conception than the Swedish and German series, so the comparison is partly between different exposure distributions rather than between different effects. A null result under these conditions is compatible with, rather than contradictory to, the pooled estimates, and the two should not be given equal weight. More generally, the conflicts in this literature cluster around three sources: differences in the interval to conception, differences in the comparator used (general obstetric population, age-matched controls, or survivors who did not conceive), and differences in how preterm birth and SGA are defined. Statistical heterogeneity in the pooled analyses is substantial for the interval-dependent outcomes and modest for congenital anomalies, which is consistent with a real biological signal concentrated in early conception and a genuinely null effect on malformation. Where studies disagree, we have given precedence to those with larger samples, population-based comparators, and explicit adjustment for the interval since diagnosis. The principal findings of the obstetric and neonatal literature are summarized in Table 1.

### 3.3. Practical Guidance on the Timing of Conception

Because timing is the variable clinicians are asked about most often, it is worth setting out how the evidence above translates into a consultation. Three separate questions are usually conflated and are better kept apart: when it is biologically possible to conceive, when it is oncologically reasonable to interrupt or complete treatment, and when the obstetric risk profile has settled.

During active treatment, the position is unambiguous. Conception should be avoided throughout chemotherapy and for the duration of endocrine, anti-HER2, PARP-inhibitor, CDK4/6-inhibitor, and checkpoint-inhibitor therapy, all of which are either teratogenic or of unknown fetal safety. Effective non-hormonal contraception should be discussed at the first treatment visit and documented, because unintended pregnancy during adjuvant therapy is the scenario with the clearest potential for harm. Washout intervals differ by agent and are frequently overlooked: tamoxifen requires approximately three months, trastuzumab seven months, olaparib six months, talazoparib seven, and pembrolizumab at least four months after the final dose [33].

After treatment, pregnancy timing should be individualized based on oncologic risk, treatment status, and reproductive factors, rather than on a fixed waiting interval [34]. Higher-risk features, including node-positive or stage III disease and substantial residual disease after neoadjuvant therapy, may favor delaying conception [26,35]. Ongoing adjuvant therapy also generally requires postponement of pregnancy, including abemaciclib for 2 years or olaparib for 1 year in eligible patients [36,37]. Conversely, node-negative stage I disease, increasing maternal age, and diminished ovarian reserve may favor earlier reproductive planning; AMH and AFC can help assess remaining ovarian reserve, although they should not be interpreted as standalone measures of fertility [10]. When these factors conflict, individualized shared decision-making among oncology, reproductive medicine, and maternal–fetal medicine is appropriate, with the rationale documented and revisited as clinical circumstances evolve [10]. For women with hormone receptor-positive disease considering interruption of endocrine therapy, the POSITIVE protocol offers a defensible template: at least 18 to 30 months of endocrine therapy completed, a three-month washout before attempting conception, interruption limited to approximately two years, and endocrine therapy resumed after delivery or after breastfeeding ends [26,27]. Once pregnancy is established, surveillance should likewise be individualized according to prior treatment and maternal risk. Women with previous anthracycline exposure, particularly those with additional cardiotoxic treatment such as trastuzumab or chest-wall radiotherapy, may require baseline and, when indicated, repeat cardiac assessment during pregnancy [38]. Given reported associations between previous cancer treatment and fetal growth restriction or small-for-gestational-age birth, third-trimester growth surveillance should be considered, particularly in women with higher-risk treatment histories [24,38]. Previous breast cancer alone does not constitute an indication for cesarean delivery; mode of birth should be based on obstetric indications, with vaginal delivery appropriate for most women [24,38].

### 3.4. Neonatal Outcomes

Parents commonly ask about the infant before they ask about themselves, and here the evidence is reassuring. The literature relevant to this question divides into two bodies of work that are not interchangeable. Direct evidence comes from infants conceived after the completion of breast cancer treatment; indirect evidence comes from infants exposed to chemotherapy in utero because the mother’s cancer was diagnosed and treated during gestation. The second group addresses a different clinical question, involves a different exposure at a different developmental stage, and carries less evidentiary weight for the counseling considered here. We treat the two separately below and label them accordingly.

Turning first to the direct evidence, neither the 2021 meta-analysis nor the 2025 meta-analysis by Song and colleagues detected a significant excess of congenital anomalies [4,24]. In *BRCA*-specific cohorts, anomalies were uncommon: 1.8% in the 2020 series and 0.9% of infants with available data in the 2024 cohort [18,28]. Preterm birth is the outcome that recurs, and its reported frequency varies widely; Azizi and colleagues documented rates from 1.2% to 21.1% across the studies they reviewed, a spread that reflects heterogeneity in cohort composition and outcome definition rather than genuine biological variability [39]. What the direct evidence does not yet provide is follow-up beyond the neonatal period. No cohort has tracked children conceived after breast cancer treatment into school age with systematic neurodevelopmental, cardiac, or metabolic assessment, and this remains the single largest gap in the field.

Longer-term data on the children are sparse, and what exists comes almost entirely from the indirect literature on cancer treated during pregnancy. We summarize it here because clinicians are asked about it, and because it is the only long-term evidence available, but its transferability to conception after treatment is limited and should be made explicit when it is used in counseling [40]. The PETALE study followed 70 children with prenatal chemotherapy exposure and found no significant excess of central nervous system, cardiac, or auditory morbidity, and no impairment of general health or growth, when assessed at 18 months or older against general population norms [41]. A 2024 population-based cohort reached a similar conclusion by a different route, attributing severe neonatal morbidity after chemotherapy in pregnancy largely to prematurity itself, with no increase in neurodevelopmental disorders or pediatric complex chronic conditions [42]. The broader cancer-in-pregnancy literature reports no consistent increase in congenital anomalies, although preterm delivery remains common [43]. The extrapolation only works in one direction, and it happens to be the reassuring one. If a fetus directly exposed to cytotoxic drugs during organogenesis shows no detectable excess of structural or neurodevelopmental harm, then conceiving months or years after those drugs have cleared is unlikely to cause harm either. This is reasoning from the greater case to the lesser one, not direct evidence, and it should be presented that way. Taken together, neonatal morbidity after breast cancer appears to be driven by prematurity rather than by malformation [24,39,42]. The distinction has practical value in the clinic, because prematurity is partly modifiable through timing and obstetric management whereas congenital anomaly risk would not be. The caveat is a familiar one: the neurodevelopmental follow-up studies are small, heterogeneous, and short, and continued longitudinal follow-up of offspring is warranted.

### 3.5. Fertility Preservation and Reproductive Health

ASCO and ESMO both recommend that fertility preservation be raised early [10,15,34]. The 2025 ASCO update classifies embryo cryopreservation, oocyte cryopreservation, and ovarian tissue cryopreservation as established options in females, and recommends referral to a reproductive specialist for any patient who is interested or undecided [10]. The reclassification of ovarian tissue cryopreservation represents the substantive change from earlier guidance, which had described the technique as evolving rather than established [9,10].

Controlled ovarian stimulation with subsequent oocyte or embryo cryopreservation remains the best-characterized strategy in women with breast cancer [13]. Modified protocols incorporating letrozole or tamoxifen were developed specifically to limit peak estradiol exposure in hormone-sensitive disease, and recent reviews describe them as feasible in routine practice [13,44]. Across systematic reviews, neither controlled ovarian stimulation nor fertility preservation more broadly has been associated with increased recurrence or with compromised survival [13].

Gonadotropin-releasing hormone (GnRH) agonists occupy a narrower but genuine role. The 2018 individual patient-level meta-analysis of five randomized trials found that concurrent GnRH agonist administration during chemotherapy reduced premature ovarian insufficiency from 30.9% to 14.1% and nearly doubled post-treatment pregnancies, from 5.5% to 10.3%, without detriment to disease-free or overall survival [45]. Guidelines are consistent on the interpretation: GnRH agonists are an adjunct, not a substitute for embryo or oocyte cryopreservation, and are most useful where cryopreservation is not feasible [9,10,46,47,48,49].

The evidence is not uniformly mature across techniques. Embryo and oocyte cryopreservation rest on a solid base that current international guidance reflects; ovarian tissue cryopreservation, GnRH agonists, and fertility preservation specifically in *BRCA* carriers do not [12]. Most of the relevant studies are observational with short follow-up, which makes head-to-head comparison between strategies unreliable. Selection should therefore be individualized to age, ovarian reserve, tumor biology, and the anticipated oncologic regimen, rather than proceeding on an assumption of interchangeability [12].

### 3.6. Reproductive Implications of Newer Adjuvant and Targeted Therapies

Almost all the figures quoted so far come from women treated with anthracycline- and taxane-based chemotherapy, tamoxifen or an aromatase inhibitor with ovarian suppression, and trastuzumab in HER2-positive disease. That is no longer the whole of adjuvant practice for young women. The agents added over the past five years change reproductive planning in ways the current evidence cannot yet measure [33,50]. Abemaciclib is given for two years in node-positive, high-risk hormone receptor-positive disease. Animal data show no direct harm to female fertility, and an exploratory analysis of 576 evaluable young women in PENELOPE-B found no significant changes in estradiol, follicle-stimulating hormone, or anti-Müllerian hormone after a year of palbociclib added to endocrine therapy [33]. All three approved CDK4/6 inhibitors are teratogenic in animals and contraindicated in pregnancy. Whether they pass into breast milk has not been studied. So the reproductive problem here is arithmetic, not gonadotoxicity. Two years of abemaciclib on top of five to ten years of endocrine therapy pushes the earliest sensible point of conception further out, in exactly the group where POSITIVE gives the weakest support for interrupting endocrine therapy. Olaparib, given for one year to *BRCA* carriers with high-risk HER2-negative disease, raises a different worry. In mice, olaparib depleted the primordial follicle reserve by about a third and caused granulosa cell dysfunction, though the same experiments found no additive effect on chemotherapy-induced follicle loss [33,51]. There are no clinical data on ovarian reserve after adjuvant PARP inhibition. All PARP inhibitors are contraindicated in pregnancy, with washout intervals of roughly six months for olaparib and seven for talazoparib. These women are young, premenopausal by definition, and already facing risk-reducing oophorectomy in their late thirties. The case for fertility preservation before PARP inhibition rather than after is therefore strong, even though the clinical evidence for gonadotoxicity is missing rather than negative. Pembrolizumab, now standard for stage II–III triple-negative disease, is the least understood of the three. Its effect on ovarian function and on pregnancy is undefined. What we know comes from preclinical work, case reports, and pooled trial safety data, with no dedicated study [33].

Contraception is advised for at least four months after the last dose [29]. In triple-negative disease, there is a further point: the recurrence risk peaks in the first two to three years, so the interval that minimizes obstetric risk and the one that minimizes oncologic risk happen to line up. That simplifies the conversation in this subgroup, even as it lengthens the wait [52]. Three things follow for practice. Referral for fertility preservation should come before the whole treatment sequence is settled, not just before chemotherapy, because that sequence now often means a delay of five years or more. Counseling should separate agents that damage the ovary from agents that are simply incompatible with pregnancy, because the two need different answers: cryopreservation for the first, contraception and a documented washout for the second. And the timing data in Section 3.2 should be applied to these regimens carefully, because no published cohort has yet reported obstetric or neonatal outcomes in women who conceived after adjuvant CDK4/6 inhibition, PARP inhibition, or checkpoint blockade.

### 3.7. Psychosocial and Patient-Reported Outcomes

Reproductive decision-making after breast cancer is not a purely biomedical calculation. Survivors describe decisional regret, anxiety, fear of recurrence, the influence of relationship status, and, repeatedly, inadequate counseling that was delayed or factually incorrect [53,54,55,56]. Reproductive concerns are both prevalent and persistent: estimates range from 21.75% to 80% across studies, with a pooled mean Reproductive Concerns After Cancer (RCAC) score of 55.84 [57]. The concerns cluster around three themes, namely the woman’s own health, the health of a future child, and fertility potential itself [55,57]. In a cross-sectional study of young Chinese women with breast cancer, the mean reproductive concern score was 59.96 ± 9.91, and scores were higher among younger women, women with more education, and women with a stronger desire for parenthood [55].

Fertility-related distress does not reliably resolve when treatment ends [58,59]. In the Swedish Fex-Can cohort, 54% of women reported high fertility-related distress 1.5 years after diagnosis, with risk concentrated among those who wanted future children, those without children, single women, women with anxiety, and women with lower infertility-related self-efficacy. Having undergone fertility preservation did not reduce distress [26], a finding worth stating plainly, since it undercuts the assumption that a technical solution addresses what is in part an emotional problem. Survivors reported fertility concerns comparable to those of infertile women without cancer and greater than those of healthy controls, together with poorer physical, role, and social quality of life [60].

The psychosocial evidence base is the weakest of those reviewed here. It draws predominantly on qualitative and cross-sectional work, which constrains both longitudinal assessment and causal inference. What emerges consistently, across methods and settings, is that inadequate fertility counseling and fear of recurrence shape reproductive decisions. Prospective studies using validated patient-reported outcome measures are needed before the long-term psychosocial impact of pregnancy after breast cancer can be described with any precision.

### 3.8. Breastfeeding After Breast Cancer

Breastfeeding sits at the intersection of the physical and the psychosocial, and it is the topic on which survivors most often report receiving no advice at all. Bhurosy and colleagues concluded that breastfeeding after breast cancer is possible but frequently complicated by prior surgery, radiotherapy, and endocrine therapy [19,30]. Uptake in practice is moderate: 33.0% of women with available data in the international *BRCA* cohort breastfed after delivery [18], and in the POSITIVE trial 196 of 317 women who gave birth (62%) initiated breastfeeding, of whom 103 (52.6%) continued for more than four months, with a median duration of 4.4 months [26]. Breastfeeding in that cohort was not associated with breast cancer-free interval events, although the number of events was small (HR 1.12, 95% CI 0.28–4.50; nine events) and the analysis was not powered to exclude a modest effect [26].

Several points can be made with reasonable confidence and are worth stating in the consultation. Lactation from an irradiated breast is usually reduced and sometimes absent, because radiotherapy causes lobular atrophy and ductal fibrosis; women should be told before delivery that the treated side may produce little or no milk. The untreated breast typically compensates, and unilateral breastfeeding is both feasible and sufficient for most infants. Breast-conserving surgery with a periareolar incision or extensive central excision is more likely to disrupt ductal continuity than a peripheral incision. Following mastectomy, breastfeeding from the contralateral breast is unimpaired. There is no evidence that breastfeeding increases recurrence risk, and no reason to advise weaning for oncologic reasons alone [61].

The constraints are pharmacological rather than oncologic. Breastfeeding is incompatible with tamoxifen, aromatase inhibitors, CDK4/6 inhibitors, PARP inhibitors, and checkpoint inhibitors, and the practical question in hormone receptor-positive disease is therefore how long endocrine therapy can reasonably remain interrupted after delivery. The POSITIVE experience suggests that a defined breastfeeding period of around four months, agreed in advance with the treating oncologist and followed by prompt resumption of endocrine therapy, is achievable without an observed safety signal [26,27]. Where surveillance imaging is required during lactation, ultrasound is preferred; mammography remains possible but is harder to interpret against a lactating background, and the breast should be emptied immediately beforehand.

The service implication is straightforward. Referral to a lactation specialist should be routine rather than reactive, ideally arranged in the third trimester so that expectations about supply from the treated breast are set before the infant arrives [40]. The psychological weight of being unable to breastfeed, or of breastfeeding from one side only, is consistently underestimated and merits dedicated support [54].

### 3.9. Guideline-Based Recommendations for Reproductive Planning After Breast Cancer

Given the increasing integration of reproductive care into breast cancer survivorship, current recommendations from major international oncology and reproductive-medicine organizations were compared to identify areas of consensus and remaining differences (Table 2). Although these guidelines address different aspects of reproductive and oncologic care, they broadly support individualized counseling, referral to reproductive specialists, and incorporation of reproductive goals into survivorship care.

**Table 2:** International guideline recommendations for reproductive planning after breast cancer.

Clinical Decision Point	ASCO [10]	ESMO [15,34]	NCCN [62]	ASRM [63]	ESHRE [64]
When should reproductive counseling occur?	At diagnosis and throughout survivorship	Before systemic treatment; reproductive options should be discussed early	Early discussion within multidisciplinary oncology care	Before potentially gonadotoxic treatment	Early counseling before treatment; individualized reproductive planning
Who should receive fertility/reproductive counseling?	All patients at risk of treatment-related infertility	Younger/premenopausal patients	Patients with potential fertility concerns	Patients facing gonadotoxic therapy or fertility treatment	Patients at risk of fertility impairment
Referral to reproductive specialists	Early referral when fertility preservation is relevant	Early referral encouraged	Referral when fertility preservation is desired	Reproductive endocrinology involvement recommended	Multidisciplinary referral recommended
Is pregnancy after breast cancer contraindicated?	No; reproductive goals should be incorporated into survivorship care	No; pregnancy may be considered after appropriate treatment	Individualized according to oncologic risk and treatment	Counseling should incorporate cancer prognosis and reproductive considerations	Individualized assessment of oncologic and reproductive factors
Timing of pregnancy	Individualized according to recurrence risk, treatment completed, age, and reproductive goals	Individualized; particularly important in HR-positive disease	Individualized according to tumor characteristics and treatment	Individualized reproductive planning	Individualized based on cancer treatment, ovarian reserve, age, and reproductive goals
Hormone receptor-positive disease	Pregnancy planning may be considered with appropriate oncologic counseling	Special attention to endocrine-therapy duration and interruption	Individualized risk–benefit discussion	Reproductive treatment should be coordinated with oncology	Individualized; evidence from contemporary studies should inform counseling
Temporary interruption of endocrine therapy	May be considered in selected patients; POSITIVE provides prospective evidence	May be considered in selected patients after adequate endocrine therapy	Individualized based on recurrence risk and treatment history	Not a primary focus	Emerging evidence supports individualized counseling
Endocrine therapy washout before conception	Follow current drug-specific recommendations	3-month washout is recommended before attempting conception after endocrine therapy	Follow drug-specific recommendations	Medication-specific reproductive safety considerations	Medication-specific; individualized
Resumption of endocrine therapy after pregnancy	Discuss resumption after pregnancy/breastfeeding as appropriate	Resume planned endocrine therapy after pregnancy	Individualized according to oncologic treatment plan	Not a primary focus	Individualized
Pregnancy in BRCA1/2 carriers	Genetic counseling should be considered	Individualized genetic and reproductive counseling	Genetic counseling/testing according to risk	Genetic counseling and reproductive options should be discussed	Genetic counseling and reproductive options should be considered
Breastfeeding after treatment	Individualized according to treatment and breast surgery	Generally feasible when clinically appropriate	Individualized	Not a primary focus	Individualized; counseling should consider breast surgery and treatment
Key emphasis	Integrating fertility into cancer care and survivorship	Shared decision-making and individualized pregnancy planning	Integration with the oncologic treatment plan	Reproductive treatment and fertility safety	Long-term reproductive health and fertility preservation

Abbreviations: ASCO, American Society of Clinical Oncology; ESMO, European Society for Medical Oncology; NCCN, National Comprehensive Cancer Network; ASRM, American Society for Reproductive Medicine; ESHRE, European Society of Human Reproduction and Embryology.

## 4. Discussion

Across heterogeneous designs, populations, treatment eras, and outcome definitions, one finding is stable: on the evidence currently available, pregnancy after breast cancer does not appear to compromise oncologic outcomes, and oncologic risk alone does not justify advising against it (Table 1). The qualification matters because the finding rests overwhelmingly on observational data showing that women who conceive differ systematically from those who do not. What can be said with confidence is that a clinically important increase in recurrence risk would probably have been detected by now across cohorts of this size; what cannot be said is that safety has been demonstrated. The obstetric and neonatal picture is less uniform, but its variability is patterned rather than random, in that excess risk is greatest in pregnancies conceived in the first year after diagnosis, declines through the second, and is largely indistinguishable from background thereafter.

Several implications follow. Pregnancy planning should be individualized against tumor biology, treatment history, the interval since diagnosis, and the woman’s own priorities, rather than governed by a single interval rule applied uniformly. We would go further and say that the two-year figure, which has acquired the status of a threshold in much of the secondary literature, is better retired in favor of the gradient the primary data actually describe. Fertility preservation is safe, guideline-endorsed, and time-sensitive, which places the conversation before treatment rather than after it; a referral delayed until chemotherapy has begun is a referral that has lost most of its value. In selected women with hormone receptor-positive disease, temporary interruption of endocrine therapy is now defensible within a multidisciplinary framework, and the updated POSITIVE analysis at 71 months strengthens that position [27]; as the trial enrolled a selected, largely stage I–II population that had already remained recurrence-free for 18–30 months on endocrine therapy, its results should not be extrapolated to women with high-risk residual disease or extensive nodal involvement. Pregnancies should be managed as high risk, with obstetric surveillance calibrated to the interval since diagnosis and to prior chemotherapy exposure. Breastfeeding is feasible for many women and should be supported rather than discouraged by default.

The most consequential development for this field is one the evidence has not caught up with. Adjuvant abemaciclib, olaparib, and pembrolizumab have entered routine practice for precisely the young, high-risk patients who are most likely to want children, and each extends the period during which conception is contraindicated by one to two years [33,50]. The gonadotoxicity of these agents ranges from probably minimal, in the case of CDK4/6 inhibitors, to plausible but unquantified, in the case of PARP inhibitors, to unknown, in the case of checkpoint blockade. Their teratogenicity, by contrast, is either established in animal models or assumed, so contraception during treatment and a defined washout afterwards are not negotiable. The practical effect is that a 32-year-old with node-positive hormone receptor-positive disease may now face a decade between diagnosis and a realistic attempt at conception, against a background of declining ovarian reserve. This makes pre-treatment cryopreservation more valuable than it was when the cohorts summarized in Table 1 were assembled, and it makes the timing evidence in Section 3.2 less directly applicable, since no published series has followed pregnancies conceived after these regimens. Reproductive outcomes should be collected prospectively within the ongoing adjuvant trials rather than reconstructed a decade retrospectively from now.

A final point concerns how this evidence is communicated. Relative risks dominate the literature, but patients reason in absolute terms, and a fivefold increase in an uncommon outcome is easily heard as a prohibition. Counseling that presents both, and that distinguishes what is modifiable from what is not, is more likely to support a decision the woman does not subsequently regret.

Substantial gaps remain. Long-term cardiovascular, metabolic, and neurodevelopmental outcomes in the children of breast cancer survivors are not well characterized. The optimal interval between modern therapy and conception is unknown, since almost all timing data derive from conventional chemotherapy and endocrine regimens rather than from targeted agents or immunotherapy. Risk stratification tools integrating tumor biology, germline genetics, and patient-level factors would improve counseling, and none currently exists in a form usable at the bedside. The apparent protective association between pregnancy and recurrence has no established biological explanation and may yet prove to be residual confounding, which is itself worth resolving. Fertility preservation data in underrepresented groups, including aggressive tumor subtypes and populations outside North America and Western Europe, are sparse. The psychosocial and economic consequences of interrupting endocrine therapy have not been studied prospectively. Progress on most of these questions will require sustained registry infrastructure and international collaboration rather than further single-center series.

This review is narrative rather than systematic: although the search was structured and screening was performed in duplicate, no protocol was prospectively registered, no formal risk-of-bias instrument was applied, and no quantitative pooling was undertaken. These methodological limitations may introduce selection and interpretive bias and preclude formal assessment of the certainty of the evidence; therefore, the findings should be interpreted as a qualitative synthesis of the available literature rather than as systematically synthesized evidence. Restriction to English-language publications will have excluded relevant work, particularly from East Asia and Latin America, where the epidemiology of young-onset breast cancer differs. The 2000–2026 window, while justified above, still spans several treatment eras, and the pre-2015 cohorts describe women who did not receive the agents now given to their high-risk counterparts. Finally, much of the primary evidence is retrospective and hospital-based, which favors academic centers and is likely to under-represent women with limited access to oncofertility services. These constraints do not undermine the central conclusions, which are consistent across designs and settings, but they do argue against treating any single effect estimate quoted here as precise.

## 5. Conclusions

Pregnancy after breast cancer is achievable and, on the evidence available at present, is not associated with an increased risk of recurrence; disease-free survival in women who conceive is equivalent to or better than in those who do not, including in hormone receptor-positive disease (HR 0.96, 95% CI 0.75–1.24) and in *BRCA1/2* carriers (adjusted HR 0.99, 95% CI 0.81–1.20). Obstetric and neonatal risks are elevated, and the elevation is best expressed in the terms the pooled data support: odds ratios are about 1.14 for cesarean delivery, 1.45 for preterm birth, 1.50 for low birth weight, and 1.16 for small-for-gestational-age birth, corresponding to relative increases of roughly one seventh to one half rather than to the multi-fold elevations sometimes quoted from interval-specific analyses. Congenital anomaly rates are not increased and sit at 0.9–1.8% in the largest carrier cohorts, within the expected population range. These risks are concentrated in conceptions occurring within the first one to two years of diagnosis and diminish as the interval lengthens, which makes timing the most actionable variable available to clinicians, provided it is treated as one modifier among several rather than as a fixed waiting period. The POSITIVE trial has extended these conclusions to hormone receptor-positive disease by showing that temporary interruption of endocrine therapy can be undertaken without a detectable short-term safety signal, a finding that now holds at a median follow-up of 71 months, with five-year breast cancer-free interval event rates of 12.3% against 13.2% in matched external controls [27]. Given the late recurrence pattern of this disease and the selected nature of the trial population, longer follow-up remains necessary before the question can be regarded as settled.

Integrating reproductive health into oncologic care has changed what is possible for young survivors. Early fertility preservation, multidisciplinary collaboration, and evidence-based planning allow many women to meet their reproductive goals without compromising cancer outcomes. What remains is to refine risk stratification, to establish reproductive outcomes after the newer adjuvant regimens, and to follow the offspring long enough to answer the questions that current data cannot. In the meantime, young women navigating survivorship alongside the wish for a child should receive counseling that is accurate, timely, and, on the evidence available, broadly reassuring, while remaining honest about where that evidence runs out.

## Figures and Tables

**Table 1:** Summary of studies reporting obstetric and neonatal outcomes after breast cancer.

Study (Citation)	Design, Sample Size, and Follow-Up	Obstetric Outcomes (Maternal)	Neonatal Outcomes	Key Message	Principal Limitations
Song et al., 2025 [1]	Systematic review and meta-analysis; 26 studies	↑ Cesarean delivery; ↑ preterm birth	No ↑ congenital anomalies	Pregnancy broadly safe; obstetric risk modest	Pooled from observational studies; heterogeneous outcome definitions; interval to conception not consistently reported
Lambertini et al., 2021 [9]	Systematic review and meta-analysis; 39 studies; 112,840 women with breast cancer, 7505 with a pregnancy after diagnosis; 8,093,401 general-population comparators	↑ Cesarean delivery (OR 1.14, 95% CI 1.04–1.25); ↑ preterm birth (OR 1.45, 95% CI 1.11–1.88); ↑ SGA (OR 1.16, 95% CI 1.01–1.33); ↑ LBW (OR 1.50, 95% CI 1.31–1.73)	No ↑ congenital anomalies	Feasible with careful monitoring	Retrospective source studies; healthy mother effect; adjustment for confounders varies between contributing studies
Labrosse et al., 2021 [15]	Single-center French cohort; 133 survivors contributing 197 pregnancies; median time from BC diagnosis to pregnancy 48 months	↔ Comparable obstetric outcomes	↔ Comparable neonatal outcomes	Previous therapy not associated with worse outcomes	Underpowered to detect ORs of the magnitude reported in the meta-analyses; single tertiary center; longer median interval from diagnosis to conception than in the register studies
Lambertini et al., 2024 [18]	International hospital-based *BRCA* cohort, 78 centers; 4732 carriers, of whom 659 had a pregnancy; median follow-up 7.8 years (IQR 4.5–12.6)	Favorable pregnancy outcomes; 91.0% delivered at term; 10.4% twin pregnancies	Congenital anomalies 0.9% (4/470 infants with available data)	Pregnancy not associated with worse disease-free survival in *BRCA* carriers	Retrospective; obstetric and fetal events extracted from oncology records and probably under-reported; gene-level subgroup estimates exploratory
Lambertini et al., 2020 [19]	International *BRCA* cohort; 1252 carriers, of whom 195 had a pregnancy; 10-year cumulative pregnancy incidence 19%	Favorable obstetric outcomes	Congenital anomalies 1.8%	No adverse oncologic or neonatal effects	Retrospective; superseded in size by the 2024 cohort; limited obstetric detail
Gkekos et al., 2024 [20]	Nationwide Swedish register study of singleton births 1973–2017; 926 births to survivors vs. 92,490 matched comparator births	↑ Cesarean delivery (RR 1.7); ↑ very preterm birth (RR 5.3); ↑ LBW (RR 2.7) when conception <1 year	↑ Prematurity	Risk is greatest with early conception and attenuates as the interval lengthens	Spans five decades of changing treatment; register data lack tumor and treatment detail; few events in the <1-year stratum
Scholz et al., 2024 [21]	German administrative-database cohort; 74 survivors vs. 222 matched controls	↑ SGA (OR 3.24 overall; OR 5.73 within 2 years); ↑ LBW (OR 4.57 within 2 years)	No major neonatal complications	Interval since diagnosis modifies obstetric risk	Very small exposed sample; wide confidence intervals; reliance on administrative coding; estimates unstable
Azizi et al., 2023 [22]	Systematic review without pooling; 29 studies and 7,359,828 pooled sample size	Variable obstetric risk	Preterm birth 1.2–21.1%; congenital anomalies uncommon	Neonatal outcomes overall reassuring	Marked between-study heterogeneity; no pooled effect estimates; inconsistent outcome definitions

↑, increased risk; ↔, no statistically significant difference. *BRCA*, breast cancer susceptibility gene; CI, confidence interval; IQR, interquartile range; LBW, low birth weight; OR, odds ratio; RR, relative risk; SGA, small for gestational age. Effect estimates are reported as given in the source publications; odds ratios and relative risks are not interchangeable and are labeled accordingly. Bracketed entries indicate figures by the authors from the primary sources.

## Data Availability

No new data were created or analyzed in this study. Data sharing is not applicable to this article.

## Supplementary Materials

The following supporting information can be downloaded at: https://www.mdpi.com/article/10.3390/cancers18162715/s1, Table S1. Complete database-specific search strategies.

## Abbreviations

The following abbreviations are used in this manuscript:

AMH	Anti-Müllerian hormone
ASCO	American Society of Clinical Oncology
*BRCA*	Breast cancer susceptibility gene
CDK4/6	Cyclin-dependent kinase 4 and 6
CI	Confidence interval
ER	Estrogen receptor
ESMO	European Society for Medical Oncology
GnRH	Gonadotropin-releasing hormone
HR	Hazard ratio
IQR	Interquartile range
LBW	Low birth weight
OR	Odds ratio
PARP	Poly(ADP-ribose) polymerase
RCAC	Reproductive Concerns After Cancer (scale)
RR	Relative risk
SGA	Small for gestational age
