# Supplementary material for: Pregnancy After Breast Cancer: A Narrative Review of Oncologic Safety, Obstetric and Neonatal Outcomes, and Reproductive Survivorship
Source: Cancers (Basel). 2026 Aug 21;18(16):2715. doi: 10.3390/cancers18162715 (PMC13510966; doi:10.3390/cancers18162715)
Supplement: Supplementary file 1 [file cancers-18-02715-s001.zip › cancers-4496412-supplementary.pdf]

Supplementary Table S1. Complete database-specific search strategies.

Database	Search strategy
**PubMed/MEDLINE**	**#1 Breast cancer:** "Breast Neoplasms"[MeSH Terms] OR "breast cancer"[Title/Abstract] OR "breast cancers"[Title/Abstract] OR "breast carcinoma"[Title/Abstract] OR "breast carcinomas"[Title/Abstract] OR "breast neoplasm"[Title/Abstract] OR "breast neoplasms"[Title/Abstract] OR "mammary cancer"[Title/Abstract] **#2 Pregnancy/outcomes:** "Pregnancy"[MeSH Terms] OR "Pregnancy Outcome"[MeSH Terms] OR pregnan*[Title/Abstract] OR conception[Title/Abstract] OR conceiv*[Title/Abstract] OR childbirth[Title/Abstract] OR "live birth"[Title/Abstract] OR "live births"[Title/Abstract] OR obstetric*[Title/Abstract] OR perinatal[Title/Abstract] OR neonatal[Title/Abstract] OR offspring[Title/Abstract] **#3 Fertility/reproductive health:** "Fertility Preservation"[MeSH Terms] OR "Reproductive Health"[MeSH Terms] OR fertil*[Title/Abstract] OR infertil*[Title/Abstract] OR oncofertility[Title/Abstract] OR reproduct*[Title/Abstract] OR "fertility preservation"[Title/Abstract] OR "ovarian reserve"[Title/Abstract] OR cryopreserv*[Title/Abstract] OR "oocyte cryopreservation"[Title/Abstract] OR "embryo cryopreservation"[Title/Abstract] OR "ovarian tissue cryopreservation"[Title/Abstract] OR gonadotoxic*[Title/Abstract] **#4 Breastfeeding/lactation:** "Breast Feeding"[MeSH Terms] OR breastfeed*[Title/Abstract] OR "breast feeding"[Title/Abstract] OR lactat*[Title/Abstract] **#5 Psychosocial/reproductive outcomes:** "reproductive concern"[Title/Abstract] OR "reproductive concerns"[Title/Abstract] OR "fertility concern"[Title/Abstract] OR "fertility concerns"[Title/Abstract] OR "fertility-related distress"[Title/Abstract] OR "reproductive distress"[Title/Abstract] OR "reproductive decision"[Title/Abstract] OR "reproductive decisions"[Title/Abstract] OR "reproductive decision-making"[Title/Abstract] OR "fertility counseling"[Title/Abstract] OR "fertility counselling"[Title/Abstract] OR "reproductive counseling"[Title/Abstract] OR "reproductive counselling"[Title/Abstract] OR "fear of

	recurrence"[Title/Abstract] **#6:** #2 OR #3 OR #4 OR #5 **#7:** #1 AND #6 **#8:** #7 AND ("2000/01/01"[Date - Publication] : "2026/07/01"[Date - Publication])
**Embase**	**#1 Breast cancer:** 'breast cancer'/exp OR 'breast cancer':ti,ab,kw OR 'breast cancers':ti,ab,kw OR 'breast carcinoma':ti,ab,kw OR 'breast carcinomas':ti,ab,kw OR 'breast neoplasm':ti,ab,kw OR 'breast neoplasms':ti,ab,kw OR 'mammary cancer':ti,ab,kw **#2 Pregnancy/outcomes:** 'pregnancy'/exp OR 'pregnancy outcome'/exp OR pregnan*:ti,ab,kw OR conception:ti,ab,kw OR conceiv*:ti,ab,kw OR childbirth:ti,ab,kw OR 'live birth':ti,ab,kw OR obstetric*:ti,ab,kw OR perinatal:ti,ab,kw OR neonatal:ti,ab,kw OR offspring:ti,ab,kw **#3 Fertility/reproductive health:** 'fertility preservation'/exp OR 'reproductive health'/exp OR fertil*:ti,ab,kw OR infertil*:ti,ab,kw OR oncofertility:ti,ab,kw OR reproduct*:ti,ab,kw OR 'fertility preservation':ti,ab,kw OR 'ovarian reserve':ti,ab,kw OR cryopreserv*:ti,ab,kw OR 'oocyte cryopreservation':ti,ab,kw OR 'embryo cryopreservation':ti,ab,kw OR 'ovarian tissue cryopreservation':ti,ab,kw OR gonadotoxic*:ti,ab,kw **#4 Breastfeeding/lactation:** 'breast feeding'/exp OR breastfeed*:ti,ab,kw OR 'breast feeding':ti,ab,kw OR lactat*:ti,ab,kw **#5 Psychosocial/reproductive outcomes:** 'reproductive concern':ti,ab,kw OR 'reproductive concerns':ti,ab,kw OR 'fertility concern':ti,ab,kw OR 'fertility concerns':ti,ab,kw OR 'fertility-related distress':ti,ab,kw OR 'reproductive distress':ti,ab,kw OR 'reproductive decision-making':ti,ab,kw OR 'fertility counseling':ti,ab,kw OR 'fertility counselling':ti,ab,kw OR 'reproductive counseling':ti,ab,kw OR 'reproductive counselling':ti,ab,kw **#6:** #2 OR #3 OR #4 OR #5 **#7:** #1 AND #6. Publication date: 1 January 2000–1 July 2026.
**Cochrane Library**	**#1:** MeSH descriptor: [Breast Neoplasms] explode all trees **#2:** ("breast cancer" OR "breast cancers" OR "breast carcinoma" OR "breast carcinomas" OR "breast neoplasm" OR "breast neoplasms" OR "mammary cancer"):ti,ab,kw **#3:** #1 OR #2 **#4:** MeSH descriptor: [Pregnancy] explode all trees **#5:** MeSH descriptor: [Pregnancy Outcome] explode all trees **#6:** (pregnan* OR conception OR conceiv* OR childbirth OR "live birth" OR obstetric* OR perinatal OR neonatal OR offspring):ti,ab,kw **#7:** MeSH descriptor: [Fertility Preservation]

	explode all trees **#8:** MeSH descriptor: [Reproductive Health] explode all trees **#9:** (fertil* OR infertil* OR oncofertility OR reproduct* OR "fertility preservation" OR "ovarian reserve" OR cryopreserv* OR "oocyte cryopreservation" OR "embryo cryopreservation" OR "ovarian tissue cryopreservation" OR gonadotoxic*):ti,ab,kw **#10:** MeSH descriptor: [Breast Feeding] explode all trees **#11:** (breastfeed* OR "breast feeding" OR lactat*):ti,ab,kw **#12:** ("reproductive concern" OR "reproductive concerns" OR "fertility concern" OR "fertility concerns" OR "fertility-related distress" OR "reproductive distress" OR "reproductive decision-making" OR "fertility counseling" OR "fertility counselling"):ti,ab,kw **#13:** #4 OR #5 OR #6 OR #7 OR #8 OR #9 OR #10 OR #11 OR #12 **#14:** #3 AND #13. Publication date: January 2000–July 2026.
